# Venezuela and its rising vector-borne neglected diseases

**DOI:** 10.1371/journal.pntd.0005423

**Published:** 2017-06-29

**Authors:** Peter J. Hotez, María-Gloria Basáñez, Alvaro Acosta-Serrano, Maria Eugenia Grillet

**Affiliations:** 1 Department of Pediatrics, National School of Tropical Medicine, Baylor College of Medicine, Houston, Texas, United States of America; 2 Department of Molecular Virology and Microbiology, National School of Tropical Medicine, Baylor College of Medicine, Houston, Texas, United States of America; 3 Texas Children’s Hospital Center for Vaccine Development, National School of Tropical Medicine, Baylor College of Medicine, Houston, Texas, United States of America; 4 Department of Biology, Baylor University, Waco, Texas, United States of America; 5 James A Baker III Institute for Public Policy, Rice University, Houston, Texas, United States of America; 6 Scowcroft Institute of International Affairs, The Bush School of Government and Public Service, Texas A&M University, College Station, Texas, United States of America; 7 Department of Infectious Disease Epidemiology, Faculty of Medicine (St. Mary’s campus), Imperial College London, London, United Kingdom; 8 Department of Parasitology, Liverpool School of Tropical Medicine, Liverpool, United Kingdom; 9 Department of Vector Biology, Liverpool School of Tropical Medicine, Liverpool, United Kingdom; 10 Laboratorio de Biología de Vectores y Parásitos, Instituto de Zoología y Ecología Tropical, Facultad de Ciencias, Universidad Central de Venezuela, Caracas, Venezuela

Poverty remains the overriding social determinant for the neglected tropical diseases (NTDs), but over the last several decades, we have also seen how political destabilization or even outright conflict can hasten economic declines and promote a substantial uptick in NTD incidence and prevalence [1]. Recent examples include the emergence of Ebola virus infection in West Africa [2], visceral leishmaniasis and other NTDs in East Africa [3, 4], and cutaneous leishmaniasis in the Middle East and North Africa [5], as well as guerilla activities linked to the drug trade in Latin America [6]. Vector-borne (taken here to encompass diseases transmitted by arthropods or snails) and zoonotic NTDs have been disproportionately represented among these emerging or reemerging infections.

A recent example of vector-borne NTDs reemerging due to political destabilization and economic collapse has been happening in Venezuela (Fig 1) [7–10].

**Fig 1:**
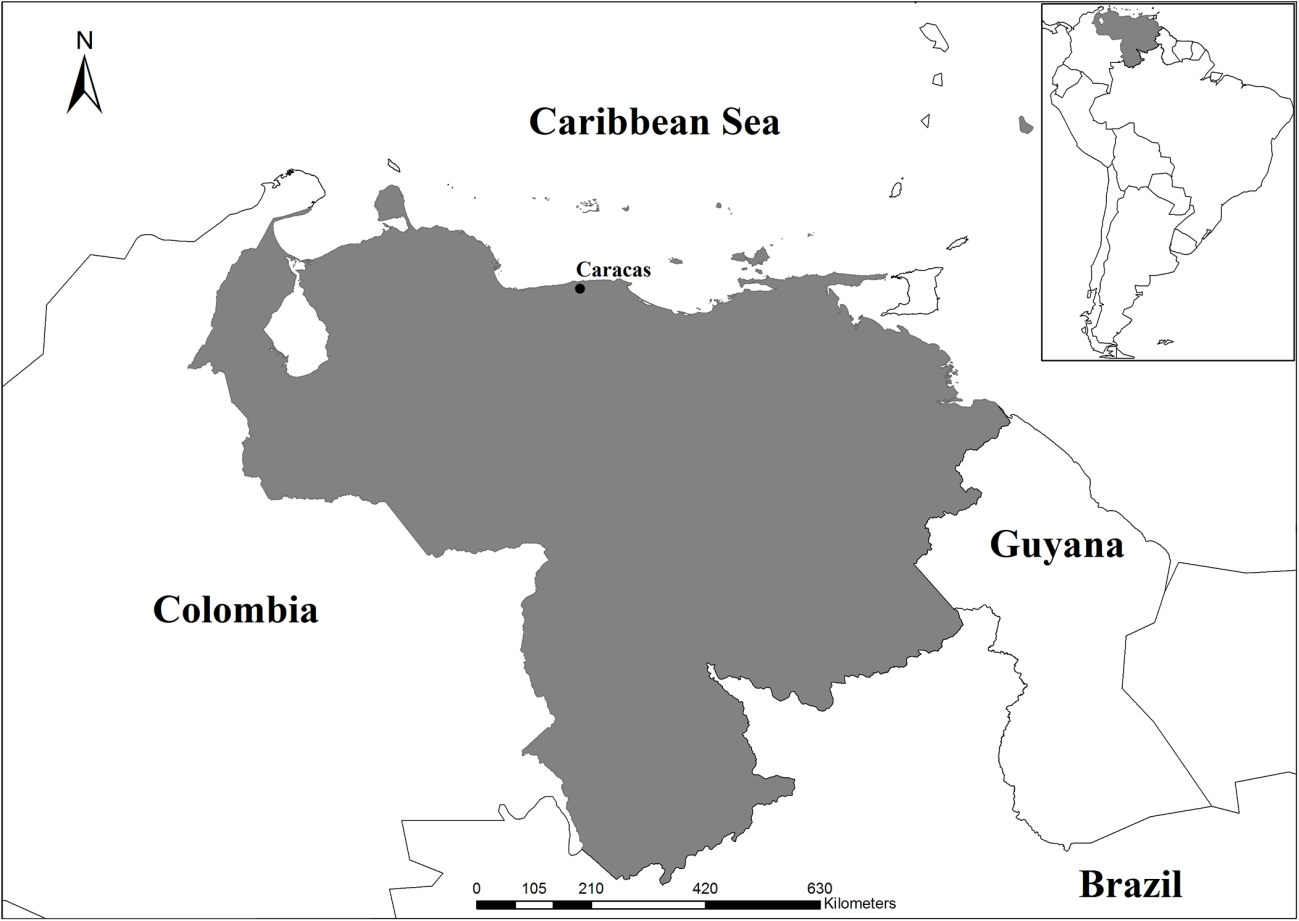
Venezuela. Original figure made by Prof. Maria Eugenia Grillet with ArcGis.

The Bolivarian Republic of Venezuela has a population of approximately 31 million with a geographic area of approximately 350,000 square miles, about 15% and 30% larger than Texas, respectively. For decades, Venezuela was considered a leader in the areas of vector control and public health policies in the Latin American region [8]. In fact, it is regarded as the first country to eradicate malaria in much of its territory after a successful insecticide-spraying campaign led by Arnoldo Gabaldón in the 1950s [11]. However, investments both in healthcare infrastructure and public health prevention efforts began to decrease during the regime of President Hugo Chavez in the 2000s, with even steeper declines beginning in 2013 with President Nicolas Maduro [8]. According to a recent report in *The Guardian*, Venezuela is currently suffering an economic crisis of historical proportions with marked negative economic growth and high rates of inflation and unemployment [12]. Food insecurity is rampant and there are serious shortages of medicines and insecticides [8,12,13]. The detailed underlying basis of Venezuela’s crisis is beyond the scope of this article, but among the major factors cited are the overreliance on oil in the setting of a crash in oil prices, “reckless” public financing and printing of public money leading to an overvalued currency, massive debts and inflation, withdrawals in external investments and donor support, and public corruption [12–14].

The political determinants of health inequity have been comprehensively reviewed by a high-level Lancet–University of Oslo Commission on Global Governance for Health [15]. Although written before the current Venezuelan crisis, many of its findings and tenets, as described below, are relevant to the current situation. Overall, the rise in Venezuela’s vector-borne NTDs can be attributed to a number of factors, including (1) shortages of insecticides, antiparasitic medicines (e.g., antimalarials and drugs for leishmaniasis and Chagas disease), and fuel, which hinder vector-control and disease-treatment efforts, (2) underlying malnutrition due to food insecurity, (3) human migrations associated with illegal mining, and (4) lapses in support for government health workers [8]. The added depletion of the health system in Venezuela due to economic and political pressures has also reduced epidemiological surveillance and reporting activities. Sadly, these determinants are playing out in the setting of an already unprecedented rise of arbovirus infections currently emerging across the Americas, including dengue, chikungunya, and Zika virus infections [1]. We have also seen widespread increases in Venezuela’s vector-borne parasitic infections.

## Vector-borne protozoan infections

Malaria caused both by *Plasmodium falciparum* and *P*. *vivax* represents 1 of the most obvious rises in Venezuela’s neglected diseases. It has been noted that highly malaria-endemic nations, such as Brazil, Colombia, and, indeed, the Americas overall, have achieved a 50% or more decrease in malaria cases [9] in pursuit of Millennium Development Goals and targets and as part of their Global Fund to Fight AIDS, Tuberculosis, and Malaria objectives and targets as they pertain specifically to the Global Malaria Action Plan 2008–2015.

In contrast, there has been almost a 3-fold increase in Venezuelan malaria cases since 2014. According to the Pan American Health Organization–World Health Organization (PAHO-WHO), there were 240,613 confirmed malaria cases in Venezuela in 2016 [16]. Among the factors fueling malaria’s rise have been illegal mining operations (especially gold mining) in the southern part of the country, with an influx of migrant workers living in overcrowded and unhealthful conditions [7, 8], together with overall shortages of essential antimalarial drugs [9], in addition to the lapses in vector-control efforts highlighted above. However, it is also possible that El Niño/La Niña–Southern Oscillations could simultaneously be fueling a component of this uptick in both *P*. *falciparum* and *P*. *vivax* malaria cases [17, 18].

During the first decades of the 20th century, Venezuela experienced 1 of the worst malaria death rates in the Americas, possibly even causing substantial population decreases [11]. Subsequently, and as noted above, Venezuela pursued an aggressive program of malaria control up until the 1970s, with dramatic decreases in malaria incidence [11]. While some gains are continuing among some Amerindian communities in Venezuela [19], overall, there are concerns that, left unchecked, an expansion in malaria cases and incidence could reverse many of the 20th century gains in malaria control. For example, it is believed that the current Venezuelan epidemic is exporting cases to Brazil, Guyana, and Colombia and creating cross-border issues with these countries. Malaria mortality will likely increase in Venezuela, which, according to a public letter (“Carta Pública”) written by 4 former health ministers, is suffering from possibly its worst malaria epidemic in the last 75 years [20].

Leishmaniasis and Chagas disease are 2 other vector-borne protozoan infections of concern. With regards to the former, there have also been shortages of antimony-containing antileishmanial drugs [9]. While some leishmaniasis research is still continuing in the country [21, 22], our overall understanding is that it has been extremely challenging to conduct research due to lack of funds and collapsed scientific infrastructure.

For Chagas disease in Venezuela, 1 of the most unusual recent features has been new urban and peri-urban outbreaks of orally acquired and food-borne disease [23–29]. Fruit juices are a common food source, especially juices contaminated with the vector *Panstrongylus geniculatus* [29]. Among the factors associated with the rise in food-borne Chagas disease may be the urbanization and deforestation of woodland areas contaminated with triatomines, the urbanization of *P*. *geniculatus*, and the higher infectivity of *Trypanosoma cruzi* (and severity of disease) noted to occur via the oral route [29]. Food-borne Chagas disease is not restricted to Venezuela, but it appears that the largest numbers of outbreaks are being reported from there.

Beyond food-borne transmission, there has also been a dramatic uptick in vector-transmitted Chagas disease. In the decades prior to the 2000s and through active surveillance, household spraying with residual insecticides, and home improvement, dramatic reductions occurred in the seroprevalence of human Chagas disease, including reductions in pediatric seroprevalence, which confirmed the impact of public health control measures on interrupting transmission [30, 31]. However, due to subsequent vector control and health system lapses during the Chavez era, the vectoral transmission and disease incidence of Chagas disease returned in both rural [32, 33] and urban areas, including Caracas [27].

Ultimately, while there is an increased research focus in oral Chagas disease transmission in Venezuela, there is also absence of political will to vigorously address this situation, despite reports being regularly made available to local and national health authorities.

## Intermediate host-borne and vector-borne helminth infections

Schistosomiasis and onchocerciasis represent 2 of the major helminth infections in Venezuela. Venezuela, Brazil, Suriname, and Saint Lucia account for the last 4 nations in the Americas with a significant level of transmission of intestinal schistosomiasis caused by *Schistosoma mansoni* [34]. In Venezuela, most of the cases are found in the northern coastal part of the country [34, 35]. Mass drug administration with praziquantel has had an impact on reducing the prevalence in many endemic communities [36], but it is believed that, in some areas, there has been an increase in schistosomiasis transmission due to depletion of surveillance and control activities [35], while urban foci of schistosomiasis may also remain [37].

Similarly, through mass drug administration with ivermectin beginning in 2000, interruption of onchocerciasis has been achieved among the northern foci located in the coastal mountain area [38, 39]. However, a southern focus in the Amazon rainforest region remains, mostly among the Yanomami indigenous groups [40]. This focus may represent 1 of the last major river blindness endemic areas in the Americas [40]. It was reported in 2016 that significant progress has been achieved recently in reducing transmission and effecting “morbidity suppression” in 75% of the Yanomami populations in this region [40]. Thus, paradoxically, the onchocerciasis elimination program in the Amazonian focus has continued to work reasonably well under the regional auspices and support of the Onchocerciasis Elimination Program for the Americas (OEPA) and from the commitments and resolve of its local health workers and managers [40].

## Arbovirus infections

Dengue, chikungunya, and now Zika virus infections are on the rise in Venezuela. Aside from lapses in vector control, other major factors include specific decreases in dengue government funding and hoarding of stored water due to lack of regular access to clean and safe water supplies [8, 41]. According to PAHO-WHO, the number of reported cases is still far below those of other Latin American countries including Brazil, Colombia, and Paraguay [41], although this finding may reflect a significant level of underreporting. Dengue remains an important cause of illness and death among Venezuelan children [42], but patient knowledge about the disease and health-seeking behavior is lacking [43]. It is believed that dengue fever was exported from Venezuela to ignite a dengue outbreak in Madeira, Portugal, in 2012–2013 [44].

In 2014, chikungunya was introduced in Venezuela, where it has also emerged as an important arbovirus infection with life-threatening and even fatal cases reported [45, 46]. Like dengue, the disease is probably vastly underreported—when it first entered the population, the attack rate may have reached as high as 13.8% [46]. It is believed that Venezuela has fallen behind in control efforts for these 2 arbovirus infections [47]. In addition, Venezuela now hosts the third largest number of Zika cases in the Americas (behind Brazil and Colombia), according PAHO-WHO [48], and the disease has become widespread in the country [49]. There are also concerns about the emergence or re-emergence of Mayaro virus [50] and Venezuelan equine encephalitis [51].

## Concluding comments

There continues to be progress in the reduction and control of Venezuela’s vector-borne helminth infections, but the abrupt rise of malaria, leishmaniasis, Chagas disease, and arbovirus infections presents a concerning and troubling situation that may be reaching crisis proportions, with spread of these diseases now occurring from Venezuela to adjacent countries. Further adding to the Venezuelan crisis, there have been attacks and vandalism on the nation’s scientists and institutions. For example, the Instituto de Medicina Tropical of the Universidad Central de Venezuela, a leading center of excellence in the Americas, has suffered repeated episodes of vandalism, with destruction of scientific equipment, records, and samples, forcing it to suspend operations. Our understanding is that this institution may not be the only one affected in Venezuela.

The Lancet–University of Oslo Commission on Global Governance for Health points out that health inequities such as those described must be addressed at the global level and not only at the national level or only within the health sector [15]. For those reasons, we urge regional leaders, possibly through the Organization of American States (OAS), together with the major UN agencies (including PAHO-WHO) and international funding organizations, to offer assistance in order to prevent a humanitarian catastrophe in the country, as well as one that could expand across the tropical regions of the Americas. Over time, the leadership of Venezuela will need to expand its efforts with the international community to restore laboratory and disease-surveillance capacity, while implementing disease-control efforts in programs of health-system strengthening.
